# Icariin Modulates the Reproductive-Immune Axis: Molecular Insights and Therapeutic Potential

**DOI:** 10.3390/cimb48040366

**Published:** 2026-04-01

**Authors:** Rongyu Wang, Yan Chen, Qinwen Xiao, Lirong Tang, Nannan Zhang

**Affiliations:** 1 Department of Traditional Chinese Medicine, West China Second University Hospital, Sichuan University, Chengdu 610041, China; rongyu-wang@scu.edu.cn (R.W.);; 2 Key Laboratory of Birth Defects and Related Diseases of Women and Children, Sichuan University Ministry of Education, Chengdu 610041, China; 3 Reproductive Endocrinology and Regulation Laboratory, West China Second University Hospital, Sichuan University, Chengdu 610041, China; 4 Department of Obstetrics and Gynecology, West China Second University Hospital, Sichuan University, Chengdu 610041, China; 5 National Center for Birth Defect Monitoring, West China Second University Hospital, Sichuan University, Chengdu 610041, China

**Keywords:** reproduction, immunology, macrophage, inflammasome, cytokine icariin

## Abstract

The immune system is a pivotal regulator of reproductive physiology, maintaining tissue homeostasis essential for successful pregnancy while contributing to infertility and reproductive disorders when dysregulated. Natural products represent a valuable source of novel immunomodulatory agents. Icariin (ICA), a prenylated flavonoid glycoside isolated from Epimedium species (Horny Goat Weed), has a long-standing traditional use for “invigorating yang,” which modern research attributes to its reproductive function-enhancing properties. This review synthesizes emerging evidence that the beneficial effects of ICA on female and male reproductive health are primarily mediated through its sophisticated immunomodulatory actions on the reproductive–immune axis. We systematically dissect the molecular mechanisms by which ICA reprograms the reproductive immune microenvironment, focusing on its regulation of macrophage polarization, T-helper cell (Th1/Th2/Th17) and regulatory T-cell (Treg) balance, and suppression of key pro-inflammatory signaling pathways (NF-κB, NLRP3 inflammasome, JAK-STAT) in ovarian, uterine, and testicular tissues. This review provides a detailed account of how ICA modulates reproductive disorders via regulating immune responses, with the aim of offering innovative strategies for the design of novel immunomodulatory therapies targeting reproductive diseases.

## 1. Introduction

Reproductive health relies on the structural and functional integrity of the reproductive system, precise endocrine signaling, and systemic homeostasis. Reproductive endocrine disorders impose a substantial global burden and severely impair quality of life. Accumulating evidence indicates that immune system dysregulation is a common pathogenic mechanism underlying these disorders. For instance, endometriosis is characterized by an imbalance between pro-inflammatory and anti-inflammatory immune cell subsets in the eutopic endometrium. Studies have shown an elevated M1/M2 macrophage ratio [1] and a reduced Treg/Th17 ratio [2] in patients with endometriosis, indicating a chronic inflammatory state within the local immune microenvironment, which is closely associated with impaired endometrial receptivity and infertility [3,4]. Similarly, polycystic ovary syndrome (PCOS), another prevalent reproductive endocrine disorder, is closely associated with immune dysregulation. Hyperandrogenism, a hallmark of PCOS, induces tissue-specific alterations in immune cell profiles within reproductive and metabolic tissues. In an androgen-exposed PCOS-like mouse model, the uterus exhibits a drastic reduction in eosinophils and an increased frequency of NK cells, accompanied by elevated IFN-γ and TNF-α levels; ovaries display a decreased macrophage population; and visceral adipose tissue shows distinct immune cell subset changes associated with insulin resistance [5]. Disruption of this immunoendocrine equilibrium impairs immune surveillance and homeostatic regulation, triggers abnormal immune cell activation, amplifies inflammatory signaling, and ultimately accelerates reproductive disease progression. Unlike isolated endocrine organs, the ovaries, testes, and uterus lie within a highly integrated immune-privileged reproductive microenvironment [6,7], whose stability is critical for normal gonadal function, periodic tissue remodeling, hormone signaling, and all reproductive stages [8]. Given the crosstalk between immunity and inflammation, anti-inflammatory and immunotherapeutic approaches have become important strategies for reproductive diseases. In reproductive health, chronic inflammation and immune imbalance drive both gender-specific reproductive disorders and reproductive aging, especially ovarian aging, which features inflammaging characterized by elevated pro-inflammatory cytokines, profibrotic macrophage polarization, pro-inflammatory T cell accumulation, and interstitial fibrosis [9,10,11]. Therefore, immune homeostasis serves as a critical therapeutic target and a key determinant of reproductive lifespan.

Despite the crucial role of immune regulation in reproductive health, traditional immunomodulatory therapies have limitations of tissue specificity and adverse effects, and traditional empirical immunotherapies are also restricted in clinical application due to uncertain efficacy and safety concerns [12]. These challenges have driven interest in plant-derived natural products, which possess multi-target regulatory properties that align with the complexity of the reproductive–immune–endocrine axis [13,14]. Preclinical studies have shown that such compounds can improve outcomes in reproductive disease models by modulating immune cell function, cytokine secretion, and immune–endocrine crosstalk [15]. Among these, icariin (ICA), a flavonoid from Epimedium (Berberidaceae), has attracted extensive attention.

Epimedium, commonly known as “Horny Goat Weed” in Western herbalism, is a representative Chinese herbal medicine first recorded in Shen Nong Ben Cao Jing, with a long history of use in regulating reproductive endocrine function [16]. The genus Epimedium comprises over 50 species, with *E. pubescens*, *E. wushanense*, *E. brevicornu*, *E. koreanum*, and *E. sagittatum* being the primary sources for medicinal use [17]. ICA, the major bioactive flavonoid glycoside, is typically extracted from the aerial parts of Epimedium using ethanol or aqueous extraction methods, followed by chromatographic purification to achieve high purity [17,18,19]. ICA is a prenylated flavonol glycoside with a molecular formula of C_33_H_40_O_15_ and a molecular weight of 676.67 g/mol, characterized by a flavonol skeleton with a glucosyl group at C-3, a methoxyl group at C-4, a prenyl group at C-8, and a rhamnosyl group at C-7. The prenyl group at the C-8 position is considered a main active site contributing to its pharmacological effects [16]. Following oral administration, ICA undergoes sequential deglycosylation mediated by intestinal microbiota to generate multiple active metabolites, including icariside I, icariside II, and its aglycone icaritin [20,21]. Tissue distribution studies have shown that ICA distributes to various organs, including the liver, lung, spleen, heart, kidney, brain, testicle, uterus, and ovary, with distribution patterns being concentration- and sex-dependent [22].

Modern studies demonstrate that ICA exerts significant biological activities in the nervous [23], reproductive [24], and skeletal systems [25,26], with its diverse pharmacological properties closely linked to its unique structural features. This structural basis endows it with various pharmacological properties such as anti-inflammatory, antioxidative, anti-apoptotic, and immunomodulatory effects, and constitutes the core mechanism for its reproductive protective effects. Based on these pharmacological properties, preclinical studies have confirmed that ICA exerts immunomodulatory and reproductive protective effects by regulating immune cell function, interfering with the secretion of key cytokines, and targeting the signaling pathways related to immunoendocrine regulation [27,28,29]. In the reproductive system, ICA can restore the immunoendocrine homeostasis of the ovary, alleviate inflammation and hormone disorders associated with endometriosis, enhance maternal immune tolerance, and protect testicular endocrine function from immune damage, and these effects are closely related to the complexity of the reproductive microenvironment. The currently identified pharmacological mechanisms of ICA can be broadly categorized into three areas: inflammatory regulation, metabolic modulation, and endocrine control. In terms of inflammatory regulation, ICA downregulates the Nuclear Factor κB (NF-κB) signaling pathway and reduces the expression of NF-κB-mediated pro-inflammatory cytokines [30]. For metabolic modulation, ICA regulates peroxisome proliferator-activated receptors (PPARs) [31] and inhibits Sirtuin 1 (SIRT1) [32]. Regarding endocrine control, ICA inhibits Phosphodiesterase 5 (PDE5) [33] and modulates the function of the hypothalamic–pituitary–adrenal axis [34,35], in addition to activating specific signaling molecules [36]. Beyond these, accumulating evidence links ICA’s pro-reproductive effects to its ability to remodel the reproductive immune microenvironment, and its protective effects may also involve regulating tissue-resident stem cell differentiation—analogous to plant extract-mediated bone homeostasis regulation—a direction worthy of future exploration [37]. Despite these advances, a key question remains to be clarified: whether the extensive reproductive protective effects of ICA are related to its role as an integrated regulatory factor of the reproductive–immune–endocrine axis. Based on this, this review aims to systematically summarize the molecular mechanisms by which ICA regulates the reproductive–immune–endocrine axis and to integrate relevant research evidence in reproductive health. We first dissect the core molecular mechanisms by which ICA modulates innate and adaptive immunity, then detail its therapeutic applications in female and male reproductive disorders, and finally provide a systems biology perspective on ICA’s signaling network. Through this integrative perspective, we aim to establish a robust theoretical foundation for the targeted development of ICA and for novel strategies in reproductive health based on systemic homeostasis regulation.

## 2. The Immunomodulation of ICA: Core Mechanisms

### 2.1. Mastering Innate Immunity

**Figure 1:**
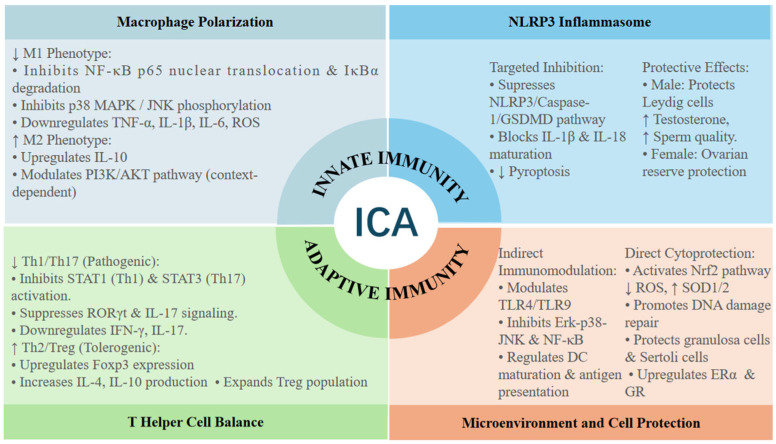
The immunomodulation of ICA.

Innate immunity serves as the first line of defense in the reproductive system, and its dysregulation is central to the pathogenesis of various reproductive disorders, including endometriosis, ovarian aging, and male infertility associated with metabolic stress [38,39,40,41]. Macrophages are the most abundant immune cells in the reproductive tract, orchestrating tissue remodeling, steroidogenesis, and immune tolerance [38,42,43,44,45], while the NLRP3 inflammasome serves as a key mediator of sterile inflammatory damage through pyroptosis, driving reproductive pathologies such as ovarian aging and testicular injury [39,41]. ICA exerts its immunomodulatory effects by targeting these core components, thereby reshaping the tissue environment to support reproductive health, as shown in Figure 1.

ICA regulates both innate and adaptive immunity through four interconnected pathways: (1) promoting macrophage M2 polarization via NF-κB/p38 MAPK inhibition; (2) suppressing NLRP3 inflammasome-mediated pyroptosis; (3) rebalancing T helper cell subsets by inhibiting Th1/Th17 and promoting Th2/Treg differentiation; and (4) modulating the immune microenvironment through TLR signaling and exerting direct cytoprotection via Nrf2 activation, including ROS reduction, upregulation of antioxidant enzymes (e.g., SOD2), promotion of DNA damage repair, and enhancement of steroid receptor signaling (ERα, GR). ↑ means increase, ↓ meanst decrease.

#### 2.1.1. Regulation of Macrophage Polarization

Chronic low-grade inflammation is a hallmark of many reproductive diseases, and ICA intervenes by targeting core effectors of innate immunity, fundamentally altering the local inflammatory state. As key resident tissue immune cells, macrophages’ polarization into pro-inflammatory (M1) or anti-inflammatory/pro-reparative (M2) phenotypes determines tissue outcomes, and the composition and function of these cells are crucial in the reproductive tract [38]. In the non-pregnant endometrium, the number of macrophages increases during the menstrual cycle and plays a role in tissue breakdown and repair [42,43]. During pregnancy, they constitute approximately 20% of decidual leukocytes and are indispensable for processes ranging from placental invasion to parturition; decidual macrophages are implicated in labor initiation, where their increased expression of inflammatory mediators promotes uterine contractions, delivery, and placental detachment [44,45].

ICA exerts a significant regulatory effect on macrophage polarization by modulating multiple key signaling pathways, primarily suppressing the M1 phenotype through inhibition of the NF-κB, p38 MAPK, and JNK pathways while promoting the M2 phenotype to facilitate tissue repair and immune tolerance. In an LPS-induced mouse model of endometritis, ICA significantly inhibited the release of pro-inflammatory factors TNF-α, IL-1β, and IL-6 and elevated levels of the anti-inflammatory cytokine IL-10 [46]. This cytokine profile shift suggests that ICA tilts the local immune balance from a pro-inflammatory M1 toward a reparative M2 phenotype by suppressing NF-κB signaling, thereby attenuating tissue damage. Similarly, in an atherosclerosis model, ICA reduced macrophage infiltration into arterial lesions, demonstrating its capacity to limit innate immune cell recruitment to inflammatory sites [47]. This anti-inflammatory property is particularly relevant to reproductive tissues, where excessive macrophage accumulation contributes to the pathogenesis of conditions such as polycystic ovary syndrome and endometriosis. Mechanistically, these effects are achieved through inhibition of pathways driving M1 polarization in LPS-stimulated macrophages. ICA derivatives suppressed phosphorylation of p38 MAPK, a key kinase that promotes production of M1-associated cytokines, supporting its therapeutic potential in conditions characterized by chronic inflammation, such as polycystic ovary syndrome and endometriosis [48].

Further studies have confirmed that ICA inhibits the NF-κB pathway, the master regulator of M1 gene expression, thereby preventing the full activation of macrophages [49,50]. However, these mechanistic findings were obtained in non-reproductive cell types such as H9c2 cardiomyocytes and rat chondrocytes, suggesting that ICA may similarly modulate NF-κB signaling in reproductive tissues, given that this pathway serves as a central inflammatory mechanism conserved across diverse cell types. In LPS-treated H9c2 cells, ICA treatment blocked c-Jun N-Terminal Kinase (JNK) phosphorylation, inhibited kappaβ inhibitor (IκBα) degradation, and prevented nuclear translocation of NF-κB p65 [49]. In rat chondrocytes, ICA similarly attenuated TNF-α-stimulated NF-κB p65 nuclear translocation and IκBα degradation, while reducing expression of IL-1, IL-6, and IL-12, as well as levels of reactive oxygen stress (ROS) and nitric oxide [50,51]. By suppressing NF-κB signaling, ICA promotes macrophage polarization toward the M2 phenotype [52] and mitigates particle-induced inflammation and osteolysis through inhibition of M1 polarization [53]. Notably, ICA-mediated regulation of macrophage phenotypes is highly context-dependent. For instance, in the aging ovary, a sustained shift toward the M2 phenotype is paradoxically associated with progressive interstitial fibrosis and functional decline [39,40]. This complexity underscores the nuanced immunomodulatory role of ICA: while promotion of a reparative phenotype may be most beneficial in acute inflammatory settings, its concomitant anti-fibrotic effects, such as potential modulation of TGF-β signaling, may be crucial for achieving favorable outcomes under chronic fibrotic conditions.

In addition to the NF-κB pathway, ICA also regulates macrophage polarization through other signaling pathways in a context-dependent manner. For example, in the tumor microenvironment, by blocking the PI3K/AKT pathway, ICA promotes macrophage conversion from the M2 to M1 phenotype, thereby inhibiting cancer cell proliferation, migration, and invasion [54]. This contrasts with the anti-inflammatory M2-polarizing effects observed in inflammatory disease models, highlighting that ICA differentially modulates macrophage polarization depending on the pathological context. In contrast, as a complex mixture containing multiple bioactive components, Epimedium extract exhibits distinct effects on macrophage regulation. Studies have shown that Epimedium extract suppresses pro-inflammatory cytokines (TNF-α, IL-1β, IL-6) while promoting the expression of TGF-β [55], suggesting that other constituents within the extract, or their combined effects, may drive macrophage polarization toward the anti-inflammatory M2 phenotype, thereby contributing to the remodeling of the inflammatory microenvironment. Moreover, M1 macrophages mainly rely on glycolysis for energy metabolism to support their rapid functional demands [56]. Upon activation, these macrophages exhibit increased levels of tricarboxylic acid cycle intermediates, including succinate, citrate, and lactate [56]. This enhancement in glycolysis, coupled with mitochondrial disruption, drives a metabolic reprogramming that shifts macrophages from a quiescent state toward a pro-inflammatory M1 phenotype, thereby triggering a more robust inflammatory response [57]. Furthermore, the upregulation of glycolytic activity in M1 macrophages elevates these metabolic intermediates and increases the expression of key glycolytic enzymes such as hexokinase II and glucose-6-phosphate dehydrogenase, further promoting the inflammatory cascade [58]. These metabolic characteristics provide a potential regulatory axis for ICA to modulate macrophage polarization and reshape the tissue immune microenvironment. Although direct evidence linking ICA to macrophage metabolic reprogramming is currently lacking, these metabolic characteristics suggest a potential regulatory axis through which ICA may modulate macrophage polarization, warranting further investigation.

#### 2.1.2. Inhibition of the NLRP3 Inflammasome

Beyond cytokine-driven inflammation, the NLRP3 inflammasome functions as a critical innate immune sensor that mediates maturation of IL-1β and IL-18 via caspase-1 activation and induces pyroptosis, contributing substantially to sterile inflammatory damage in the reproductive system, as demonstrated in conditions such as obesity-induced male infertility and ovarian aging [41,59]. ICA has been shown to directly target and inhibit this signaling axis across multiple pathological models. In a lupus nephritis model, its renal protective effects were closely associated with suppression of NLRP3 inflammasome activation and subsequent IL-1β production [60]. More directly relevant to reproductive health, in a high-fat-diet-induced obese male infertility model, ICA significantly inhibited the NLRP3/caspase-1/GSDMD pathway in testicular tissue and Leydig cells, reducing IL-1β and IL-18 expression and effectively alleviating Leydig cell pyroptosis [41]. This protection was directly associated with recovery of serum testosterone levels and improvements in spermatogenesis, including increased sperm count and quality [41]. The therapeutic relevance of this regulatory mechanism extends to the female reproductive system. Genetic evidence demonstrates that global NLRP3 inflammasome knockout significantly enhances ovarian reserve, increases anti-Müllerian hormone levels, and improves fertility outcomes in aged mice [59], confirming that inhibition of the NLRP3 pathway alone is sufficient in counteracting age-related reproductive decline. Collectively, these findings indicate that ICA protects reproductive function by inhibiting NLRP3 inflammasome-mediated pyroptosis in key reproductive cells, thereby preserving tissue integrity and endocrine function.

### 2.2. Orchestrating Adaptive Immunity

Adaptive immunity provides antigen-specific regulation essential for reproductive success, particularly at the maternal–fetal interface where immune tolerance must be precisely balanced against pathogen defense. T helper cell subsets and dendritic cells orchestrate this delicate equilibrium, and their dysregulation underlies conditions such as recurrent miscarriage and ovarian insufficiency. ICA modulates adaptive immunity through dual mechanisms: balancing T helper cell responses and shaping the antigen-presenting cell microenvironment.

#### 2.2.1. Balancing the T-Helper Cell Response

The balance of helper T cell (Th cell) subsets is the core mechanism by which ICA regulates adaptive immunity and enhances reproductive immune tolerance. This process is particularly critical in reproductive tissues, where finely tuned adaptive immune responses determine the establishment of immune privilege at the maternal–fetal interface [61,62]. Dysregulation of T cell-mediated immunity is a key driver of reproductive immune-related disorders, such as recurrent miscarriage and preeclampsia [63]. ICA functions as a master regulator of T cell fate, employing a dual mechanism that inhibits pro-inflammatory Th1 and Th17 cells while promoting anti-inflammatory Th2 and Treg cells. This action is characterized by the suppression of IFN-γ and IL-17 secretion from Th1 and Th17 cells, and the enhancement in IL-4, IL-10, and Foxp3 expression in Th2 and Treg populations. The process is primarily driven by ICA’s modulation of the JAK-STAT, RORγt, and TGF-β/Smad signaling axes.

In early pregnancy, although T cells constitute a relatively small proportion of decidual immune cells, they undergo marked functional specialization: compared to T cells in peripheral blood during the third trimester, decidual CD4+ and CD8+ T cells exhibit a highly differentiated effector memory phenotype, accompanied by increased production of IFN-γ and interleukin-4 (IL-4) [64,65]. This shift in cytokine profile helps balance pro-inflammatory and anti-inflammatory responses, thereby creating a stable immune milieu conducive to embryo implantation and development. Notably, a pathological shift toward Th1 and Th17 responses, often accompanied by impaired Th2 and Treg function, is considered an important immunological mechanism underlying immune-related reproductive failure, such as recurrent miscarriage and ovarian insufficiency, though these conditions are multifactorial in nature.

In terms of Th17 cell regulation, Jinfeng Wan, a herbal formulation containing ICA with Epimedium as the monarch drug, significantly improves ovarian function in a cyclophosphamide-induced rat model of Premature Ovarian Insufficiency (POI). Its key therapeutic mechanism is closely linked to the inhibition of the IL-17 signaling pathway and Th17 cell differentiation. In vivo experiments confirmed that Jinfeng Wan markedly downregulates IL-17A mRNA expression in ovarian tissue and its protein level in serum, while also suppressing the expression of its downstream pro-inflammatory factors IL-6 and COX-2 [66]. Additionally, it inhibits the expression of immediate early genes associated with Th17 differentiation, including FOS, FOSB, and FOSL1, as well as downstream effectors such as MMP3, MMP13, and COX-2. Mechanistically, Jinfeng Wan suppresses the phosphorylation of MEK1/2 and ERK1/2, and its effects are potentially linked to modulation of RORγt activity, a key transcription factor governing Th17 cell development, as suggested by transcriptomic and network pharmacology analyses [66].

Regarding Th1 cell regulation and transcriptional control, ICA precisely corrects Th cell subset imbalance by inhibiting the activation of STAT1 and STAT3, transcription factors that drive Th1 and Th17 differentiation respectively [67]. This mechanism is directly relevant to reproductive immunopathology: for instance, in the aging ovary, there is a significant accumulation of pro-inflammatory Th1 and Th17 cells, particularly within the CD4−CD8− double-negative subset, and the cytokines they secrete drive local inflammation and tissue dysfunction [5]. Importantly, ICA-mediated inhibition of STAT1/3 directly counteracts this age-related pathogenic shift in T cell subsets.

In the context of anti-inflammatory phenotype regulation and reproductive protection, Treg cells, especially Foxp3+ Treg subsets, are key executors of maternal–fetal immune tolerance, and the stability of their number and function is critical for successful pregnancy. Decidual Foxp3+ Tregs, along with FoxP3-negative PD-1+ or TIGIT+ CD4+ T cells, inhibit effector T cell activation to prevent maternal immune rejection of the embryo [68]. Conversely, a significant reduction in Foxp3+ Treg subsets is observed in decidual tissue from miscarriages with normal fetal chromosomes [69]. ICA enhances immune suppressive function by promoting Treg cell development and upregulating the expression of its specific marker Foxp3: in mice with recurrent spontaneous abortion (RSA), ICA increases the placental Treg cell population while reducing pro-inflammatory cells and their associated cytokines [70], thereby decreasing the spontaneous abortion rate by enhancing immune tolerance. In mice with autoimmune primary ovarian insufficiency induced by three zona pellucida peptides, ICA also increases ovarian Treg cell expression, which improves ovarian structure and function [71]. Collectively, ICA reconstructs immune homeostasis in the reproductive system through a two-pronged strategy: inhibiting Th1/Th17 differentiation at the transcriptional level and promoting Treg development, thereby safeguarding successful pregnancy and gonadal health.

#### 2.2.2. Modulation of Dendritic Cell Function and the Immune Microenvironment

Dendritic cells (DCs) act as key bridges connecting innate and adaptive immunity, and their maturation status and antigen-presenting function directly determine the direction of T cell responses [72]. Both are critical for establishing and maintaining immune tolerance in the reproductive system. Abnormal DC maturation can overactivate pro-inflammatory T cell subsets and disrupt immune balance [73]. Although direct studies on ICA effects on reproductive system DCs remain limited, its anti-inflammatory, antioxidant, and multi-target signaling regulatory properties can indirectly influence DC function and T cell responses by modulating the innate lymphoid cell microenvironment.

In the uterine decidua, 70% of immune cells in early pregnancy are decidual natural killer (dNK) cells, which are non-cytotoxic and secrete pro-angiogenic factors to promote placental formation [44,74,75,76]. ICA’s ability to reduce oxidative stress and inflammation supports this pro-remodeling function of dNK cells. Additionally, γδ T cells, enriched in the decidua during early pregnancy, produce high levels of the anti-inflammatory cytokines IL-10 and TGF-β, creating a regulatory immune environment [77,78]. ICA’s capacity to activate Nrf2 helps maintain this tolerogenic microenvironment, which is crucial for implantation and pregnancy maintenance, thereby laying a foundation for appropriate DC-mediated T cell responses.

The core molecular mechanisms by which ICA regulates immune cell function, and indirectly affects DC maturation and antigen presentation, primarily involve the modulation of TLR, Erk-p38-JNK, and NF-κB signaling pathways. In terms of TLR regulation, ICA dose-dependently induces TLR9 expression in mouse macrophages and upregulates the expression of its downstream molecules, such as IL-6 and TNF-α [79], while simultaneously reducing TLR4 expression in human peripheral blood mononuclear cells (PBMCs) [80]. Although these observations were made in macrophages and PBMCs, TLR signaling pathways are conserved across myeloid cell types. Given that TLR9 activation promotes DC maturation and antigen presentation, while TLR4 signaling is associated with pro-inflammatory DC activation, the regulatory effects of ICA on these pathways suggest a potential mechanism by which ICA may modulate DC function, though direct evidence in DCs remains to be established. This modulation of TLR signaling ultimately influences the expression of DC antigen presentation-related molecules. Regarding the Erk-p38-JNK pathway, known to regulate IL-2 secretion signals in T lymphocytes [81,82,83], ICA modulates immune cell differentiation by inhibiting the phosphorylation of Erk1/2-p38-JNK signaling molecules [84], and it also exerts anti-inflammatory effects by suppressing the activation of Erk-p38-JNK and NF-κB signaling pathways induced by IL-1β [85].

In addition to its immune regulatory effects, IICA supports reproductive homeostasis through both molecular modulation and direct cytoprotection. At the molecular level, ICA upregulates Glucocorticoid Receptor (GR) mRNA expression in depressed rats subjected to unpredictable chronic mild stress (CMS), exerting anti-inflammatory effects via crosstalk between GR and signaling pathways such as TCR and PI3K [86]. It also promotes PTEN expression in ovarian cancer A2780 cells, thereby influencing adaptive immune cell development through modulation of the PI3K pathway [87,88]. Furthermore, ICA negatively regulates the NLRP3/caspase-1/IL-1β axis in the hippocampus of CMS rats and inhibits NF-κB-mediated NLRP3 activation [30,89], reducing pro-inflammatory cytokine release and preventing reproductive tissue damage caused by abnormal immune activation. At the same time, ICA exerts direct antioxidant and anti-apoptotic effects on reproductive cells. It protects porcine oocytes from in vitro aging-induced damage by reducing ROS activity and upregulating antioxidant genes, including Superoxide Dismutase 1/2 and Nrf2 [90,91]. In a D-galactose-induced POI model, ICA enhances ovarian follicular development, elevates estradiol and anti-Müllerian hormone levels, and safeguards granulosa cells by promoting DNA damage repair [90,91,92]. Additionally, ICA mitigates age-related testicular dysfunction and protects Sertoli cells via upregulation of Estrogen Receptor alpha (ERα) and Nrf2 signaling [93], collectively providing a robust tissue foundation for maintaining immune homeostasis in the reproductive system.

## 3. Therapeutic Applications: From Molecular Targets to Disease Management

**Figure 2:**
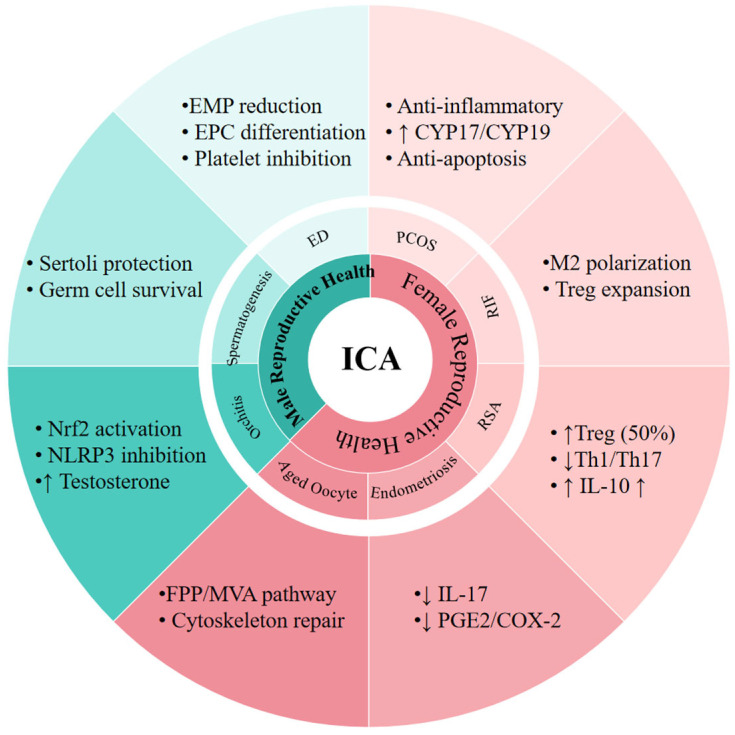
ICA therapeutic applications in reproductive disorders.

Based on the aforementioned molecular mechanisms of ICA and its compounds in regulating reproductive immune homeostasis, this section focuses on their specific therapeutic applications in reproductive system diseases, clarifying the translation process from molecular target regulation to clinical disease management, and providing practical theoretical support for clinical application, as shown in Figure 2.

ICA exerts therapeutic effects on female reproductive disorders including PCOS, RIF, RSA, endometriosis, and aged oocyte, as well as male reproductive disorders including orchitis, spermatogenesis, and erectile dysfunction, with corresponding mechanisms detailed in the peripheral grid. Abbreviations: RIF, recurrent implantation failure; RSA, recurrent spontaneous abortion; ED, erectile dysfunction; EMP, endothelial microparticle; EPC, endothelial progenitor cell; PCOS, polycystic ovary syndrome. ↑ means increase, ↓ meanst decrease.

### 3.1. Female Reproductive Health

#### 3.1.1. Ovarian Function and PCOS

PCOS is closely associated with ovarian and systemic low-grade inflammation, which represents a key pathological driver of abnormal folliculogenesis, anovulation, and reproductive endocrine disorders [94]. ICA has been shown to alleviate PCOS symptoms in letrozole- and high-fat-diet-induced rat models, with therapeutic effects mainly reflected in restored ovarian function and endocrine profiles: it regulates hormone levels, rescues the estrous cycle, and alleviates PCOS-related ovarian morphological damage [95,96]. In contrast to the T cell- and DC-oriented immune regulation observed in POI, the therapeutic effects of ICA in PCOS involve multiple mechanisms. ICA suppresses local ovarian inflammation, thereby repairing the impaired follicular developmental microenvironment. Additionally, ICA directly inhibits the ovarian IL-6/gp130/JAK2/STAT3 signaling pathway and reduces granulosa cell apoptosis, thereby improving the pathological state of PCOS in rats [95]. This direct intraovarian anti-inflammatory effect helps eliminate the inflammatory milieu that disrupts folliculogenesis and supports normal follicular development. In addition to its anti-inflammatory actions, ICA promotes ovarian granulosa cell proliferation and steroidogenesis by upregulating the expression of CYP17 and CYP19 [96], further supporting healthy follicular development and ovulatory function. Notably, PCOS and ovarian aging share overlapping pathological features, including abnormal inflammation and dysregulated macrophage activity [9], suggesting that the therapeutic mechanism of ICA in PCOS may be broadly applicable to protecting ovarian function against various inflammatory injuries, offering a potential target for multiple ovarian dysfunction-related diseases.

#### 3.1.2. Endometriosis

Endometriosis is a common chronic inflammatory gynecological disorder characterized by ectopic implantation, survival, and invasion of endometrial tissue outside the uterine cavity [97]. Its pathogenesis is closely linked to abnormal inflammatory activation and immune microenvironment dysregulation, among which the IL-17-mediated inflammatory cascade and dysregulated COX-2/prostaglandin E2 (PGE2) signaling are critical drivers. Although direct clinical evidence supporting ICA for endometriosis is limited, pharmacological studies suggest its potential in targeting these key pathways. The anti-inflammatory properties of ICA are well-documented in several experimental disease models, where it has been shown to target the IL-17 inflammatory axis. For instance, in a murine model of collagen-induced arthritis, ICA treatment suppressed Th17 cell differentiation and reduced IL-17 production by inhibiting STAT3 phosphorylation, thereby alleviating joint inflammation [98]. Similarly, in studies on experimental autoimmune encephalomyelitis (EAE), ICA not only decreased serum IL-17 levels but also synergized with methylprednisolone to enhance this effect, contributing to reduced neuroinflammation [99]. Furthermore, ICA was found to inhibit Th1 and Th17 cell differentiation via modulation of dendritic cells in the same EAE model, further validating its regulatory role on pathogenic T-cells [100]. Furthermore, PGE2, a key mediator of inflammation and tissue invasion, is frequently elevated in endometriosis and contributes to disease progression. An animal study demonstrated that Epimedium extract (a natural source of ICA) reduced serum PGE2 levels in Ovariectomized (OVX) rats in a dose-dependent manner [55], implying that ICA may act by suppressing the COX-2/PGE2 pathway and subsequent PGE2 production. Collectively, by modulating both IL-17 and PGE2-related pathways, ICA exhibits promise for a multi-target therapeutic strategy to curb the survival and invasiveness of ectopic endometrial tissue. This approach offers a novel direction for clinical management, particularly for patients presenting with significant inflammatory components.

#### 3.1.3. Endometrial Receptivity and Recurrent Implantation Failure

A core pathological mechanism of recurrent implantation failure (RIF) is immune microenvironment imbalance at the maternal–fetal interface, where M2-polarized macrophages and regulatory T cells are indispensable for maintaining immune tolerance and supporting embryonic development. ICA has been shown to specifically promote the recruitment and function of these two key immune populations at the maternal–fetal interface [70,71], offering a targeted therapeutic strategy for RIF.

Notably, LPS-induced chronic endometritis severely impairs endometrial receptivity and contributes to RIF. ICA effectively alleviates LPS-induced mouse endometritis by inhibiting the TLR4/NF-κB pathway to reduce inflammation and activating the Nrf2 pathway to counteract oxidative stress [46]. In addition, Epimedium extract has been confirmed to reduce the expression of multiple pro-inflammatory factors (including TNF-α, IL-1β, and PGE2), helping establish an immunologically tolerant maternal–fetal microenvironment and potentially improving embryo implantation success [55]. During the implantation window, polarization of endometrial macrophages toward an anti-inflammatory, tissue-repairing, and pro-angiogenic M2 phenotype is critical. M2 macrophages secrete cytokines such as IL-10 and TGF-β, which directly facilitate trophoblast invasion and spiral artery remodeling—essential steps for successful maternal–fetal circulation [75,101,102]. Research indicates that ICA effectively drives the transition of macrophages from the pro-inflammatory M1 phenotype to the M2 phenotype across various inflammatory and tissue repair contexts, with mechanisms involving suppression of TLR4/NF-κB and ERK/HIF-1α pathways [103]. Although direct evidence in endometrial tissue remains limited, these findings suggest that ICA may similarly modulate endometrial macrophage polarization, thereby potentially increasing the proportion of local M2 macrophages and improving endometrial receptivity.

Beyond promoting M2 macrophage polarization, ICA improves RIF outcomes by expanding the Treg population to establish stable maternal–fetal immune tolerance. Tregs are essential for maintaining maternal immune tolerance toward the semi-allogeneic fetus by suppressing embryo-targeting effector T cell responses (e.g., Th1, Th17 cells) at the implantation site. RIF patients often display a reduced number or impaired function of endometrial Tregs. ICA significantly promotes Treg proliferation and function [70,71], thereby enhancing active immune suppression and preventing immune rejection-mediated implantation failure.

The core immunological mechanism by which ICA improves endometrial receptivity and treats RIF is therefore a dual-targeted regulation: inducing supportive M2 macrophages to provide structural and nutritional support for embryo implantation and development, while expanding protective Tregs to shield the fetus from maternal immune attack.

#### 3.1.4. RSA

In addition to local immune imbalance at the maternal–fetal interface, chronic endometrial inflammation caused by ascending genital tract infections (such as endometritis) is also an important environmental factor leading to RSA. Such inflammatory stress disrupts maternal–fetal immune tolerance through sustained release of pro-inflammatory factors. In this pathological process, ICA shows potential in multi-target regulation of inflammation and immune microenvironment. Studies have shown that in the LPS-induced mouse endometritis model, ICA can significantly inhibit the TLR4/NF-κB signaling pathway, reduce pro-inflammatory factors (TNF-α, IL-1β, IL-6), and increase the anti-inflammatory cytokine IL-10 [46], providing direct experimental evidence for correcting Th1/Th2 and Th17/Treg imbalance in RSA and establishing a favorable uterine microenvironment for embryonic development.

Immune rejection driven by a Th1/Th17-skewed response is a major etiology of RSA. The human decidua naturally possesses a multifaceted regulatory T cell network to prevent such rejection [68]. The marked efficacy of ICA in RSA models is reflected in its active rebalancing of the maternal–fetal immune dialog: it increases protective Tregs at the placental interface while reducing pro-inflammatory Th1/Th17 cytokines [70]. This pharmacologic enhancement in natural tolerance mechanisms represents a promising therapeutic strategy to prevent miscarriage, particularly in cases associated with Treg deficiency [69].

#### 3.1.5. Improving Aged Oocyte Quality

Age-related decline in oocyte quality is central to female fertility reduction, traditionally linked to chronic low-grade inflammation and oxidative stress. However, recent studies reveal that ICA and its active derivatives directly counteract core cytological defects of oocyte aging through novel pathways largely independent of classical immune regulation. These metabolites, especially FPP, are essential substrates for farnesylation post-translational modification of small GTPases (e.g., CDC42 and RAC1), which is required for their membrane localization and activation and crucial for cytoskeleton dynamics [104]. Notably, 8-isopentenylflavone (8-IPF), a natural flavonoid derived from Epimedium and structurally related to ICA, exhibits strong protective activity. Its mechanism does not act directly on the oocyte itself, but precisely targets surrounding granulosa cells. 8-IPF treatment significantly upregulates the expression of rate-limiting enzymes in the mevalonate pathway (e.g., MVK and FDPS) in granulosa cells, thereby promoting FPP synthesis. Using innovative chemical reporter tracing, it has been confirmed that FPP synthesized in granulosa cells is actively transported into oocytes via intercellular junctions (e.g., gap junctions). Within oocytes, this exogenous FPP supports farnesylation of CDC42 and RAC1, promoting their membrane localization and activating the downstream CDC42-N-WASP-Arp2/3 and RAC1-WAVE2-Arp2/3 signaling complexes. Activation of these complexes ultimately drives the reassembly and stabilization of the cortical F-actin cytoskeleton. The functional significance of this “metabolite supply–protein modification–cytoskeleton reconstruction” axis is substantial: in aged mouse models, supplementation with MVA, FPP, or 8-IPF significantly restores oocyte cortical F-actin intensity, greatly reduces chromosome alignment errors and aneuploidy during meiosis, and ultimately improves oocyte in vitro fertilization and blastocyst formation rates, as well as pregnancy rates and litter sizes in aged female mice. This mechanism clearly demonstrates that ICA and its derivatives can act not only as “immune microenvironment regulators” but also as “germ cell metabolic support system enhancers”. By repairing the metabolic dialog between oocytes and their supporting somatic cells, they directly maintain germ cell autonomous health and functional integrity, providing a novel therapeutic target based on metabolic reprogramming and cytoskeleton engineering for clinical intervention in age-related infertility [104].

### 3.2. Male Reproductive Health

#### 3.2.1. Autoimmune Orchitis and Male Infertility

The spermatogenic microenvironment is highly susceptible to inflammation and oxidative stress, and the main role of ICA is to create and maintain a protective anti-inflammatory microenvironment. In testicular models, this effect is achieved by activating the Nrf2 antioxidant pathway in Sertoli cells, thereby protecting both Sertoli cells and germ cells from oxidative damage [93]. Additionally, ICA supports Leydig cell function and testosterone production [105], and testosterone itself possesses inherent immunomodulatory properties. By inhibiting core inflammatory pathways such as NF-κB and p38 MAPK [49,50], ICA further reduces inflammation-induced apoptosis in the testis.

These comprehensive effects contribute to the maintenance of testicular homeostasis, supporting blood–testis barrier integrity and reducing leukocyte infiltration under metabolic, toxic, or age-related challenges [106,107,108]. Epimedium extract has shown clear anti-inflammatory effects in the OVX model, reducing inflammatory mediators such as TNF-α and IL-1β [55]. This suggests a potential mechanism by which Epimedium extract might reduce leukocyte infiltration and protect the blood–testis barrier, thereby inhibiting the production of anti-sperm antibodies in orchitis, warranting further investigation.

#### 3.2.2. Spermatogenesis

Although direct autoimmune orchitis models are needed, the pharmacological characteristics of ICA make it a strong candidate drug. Its ability to inhibit NLRP3 inflammasome activation [60] and shift T cell responses toward tolerance (increasing Treg cells and inhibiting Th1/Th17 cells) [67,70,71] provides a mechanistic blueprint for calming testicular autoimmune inflammation, protecting germ cells, and potentially reducing the production of anti-sperm antibodies. Beyond its regulatory role in testicular inflammation and spermatogenesis, ICA also exerts significant therapeutic effects on other male reproductive disorders associated with the imbalance of the reproductive–immune axis, particularly vascular erectile dysfunction (ED).

#### 3.2.3. Vascular ED

Vascular ED is a common male reproductive disorder and a typical clinical manifestation of the “reproductive–immune–vascular” axis imbalance—an integrated framework that recognizes the complex interplay between endocrine, immune, and vascular systems in the pathogenesis of reproductive disorders. Its pathogenesis is closely associated with endothelial dysfunction, chronic low-grade inflammation, and a prothrombotic state [109,110,111]. Consistent with its multi-target immunomodulatory and anti-inflammatory properties observed in other reproductive diseases, ICA exerts distinct multi-target repair effects in this pathological process. In the hypertensive ED model, ICA treatment significantly reduces the level of circulating endothelial microparticles (EMPs)—markers of endothelial injury and activation that also act as pro-inflammatory mediators, capable of inhibiting the Akt/eNOS pathway and exacerbating oxidative stress [112,113]. Reducing EMP levels suggests that ICA alleviates local vascular immune-inflammatory signaling at its source. This is particularly relevant given that inflammatory mediators such as TNF-α and IL-6, which are elevated in cardiovascular and metabolic diseases, have been shown to impair cavernosal smooth muscle relaxation and downregulate eNOS expression [111,114].

Meanwhile, ICA has been shown to enhance vascular repair capacity, which may involve the regulation of endothelial progenitor cells, thereby contributing to the reversal of endothelial dysfunction [115,116]. This endothelial protective effect is especially important in the context of innate immune system activation. Previous studies have shown that activation of Toll-like receptors by damage-associated molecular patterns (DAMPs) or oxidized lipoproteins contributes to vascular inflammation and ED through the production of pro-inflammatory cytokines and Reactive Oxygen Species [117,118,119]. In terms of thrombo-inflammation regulation, ICA significantly reduces platelet activation indicators by inhibiting the PI3K-Akt pathway, including mean platelet volume, platelet distribution width, and the expression of integrin αVβ3. This effectively inhibits the pro-inflammatory and pro-aggregatory activities of platelets, and these combined effects break the vicious cycle of “endothelial injury–platelet activation–inflammatory amplification”—a key step in restoring reproductive function-related vascular homeostasis [116].

ICA therefore not only improves local hemodynamics but also systematically restores the homeostatic balance of the axis connecting reproductive function, immune homeostasis, and vascular health by synchronously regulating immune-related endothelial injury and vascular inflammation. This provides new mechanistic insights for the treatment of metabolic and inflammation-related vascular ED.

## 4. The Signaling Network of ICA: A Systems Biology Perspective

From a systems biology perspective, the regulatory effect of ICA on reproductive immune homeostasis relies on a coherent signaling network rather than single-target or linear pathway regulation, which integrates immune–inflammatory pathways (e.g., NF-κB), metabolic pathways (e.g., MVA pathway), and oxidative stress-related pathways (e.g., Nrf2/HO-1 pathway) with crosstalk and synergistic interactions. Detailed information of these key pathways is systematically summarized in Table 1.

## 5. Challenges and Future Perspectives

Despite its promising therapeutic potential, ICA faces several application bottlenecks and research limitations that hinder its translation from basic research to clinical practice. The primary challenge pertains to its poor oral bioavailability resulting from low water solubility, degradation by intestinal flora, and a pronounced hepatic first-pass effect [124,125]. Consequently, only a limited fraction reaches the systemic circulation, substantially diminishing clinical efficacy. Additionally, quality control remains difficult as ICA content in Epimedium varies considerably with geographical origin, harvesting time, and extraction methods, leading to inconsistent purity and bioactivity across studies [126]. Although standardized pharmacopeial methods, such as high-performance liquid chromatography (HPLC), have been established for the quantification of ICA in Epimedium in the Chinese Pharmacopoeia [127], their application in research settings remains inconsistent, contributing to variability in reported outcomes. Furthermore, current systemic administration lacks targeted delivery to reproductive organs such as the ovaries, testes, and endometrium, resulting in suboptimal local drug concentrations and potential adverse effects from non-specific distribution [124]. Regarding mechanistic investigations, the multi-target regulatory characteristics of ICA complicate efforts to identify critical targets and core signaling networks. Existing studies predominantly focus on individual diseases or isolated pathways, leaving synergistic relationships between immune modulation and metabolic regulation poorly characterized. Moreover, most mechanistic studies rely on in vitro or animal models with limited translational relevance to human physiology.

To address these challenges, future research should prioritize optimizing pharmacokinetic properties through structural modification and advanced delivery systems including nanocarriers and liposomes to enable organ-specific targeting [128]. Establishing standardized quality control using modern analytical techniques such as high-performance liquid chromatography is essential to ensure consistency and reproducibility [126]. For mechanistic elucidation, systems biology and multi-omics approaches should be employed to systematically map the multi-target regulatory networks of ICA and identify key nodes in reproductive immune homeostasis. Well-designed clinical trials are warranted to evaluate efficacy, safety, and optimal dosing in reproductive diseases, particularly in conditions with established immune–inflammatory pathogenesis such as RIF, PCOS, POI, and ED, where preclinical evidence for ICA is strongest. Combination strategies integrating ICA with conventional hormones or immunomodulators may harness synergistic effects to improve outcomes and reduce adverse reactions. Ultimately, addressing these priorities will facilitate the clinical translation of ICA and expand therapeutic options for reproductive medicine.

## 6. Conclusions

This review systematically summarizes the pharmacological mechanisms, therapeutic applications, existing challenges, and future research priorities of ICA in regulating reproductive immune homeostasis and treating reproductive system diseases. Current evidence indicates that ICA, as a natural bioactive compound with multi-target regulatory properties, exhibits significant potential in improving both female and male reproductive health. These effects are closely associated with its comprehensive modulatory actions on the reproductive–immune axis and non-immune regulatory pathways.

In the context of female reproduction, ICA ameliorates pathological conditions associated with PCOS, endometriosis, RIF, and RSA. The underlying mechanisms involve the establishment of a foundation for reproductive function recovery through regulation of immune and inflammatory balance. Specifically, ICA inhibits the NF-kB and p38 MAPK signaling pathways, promotes M2 macrophage polarization and regulatory T cell expansion, and reconstructs maternal–fetal immune tolerance. The effect of ICA on aged oocyte quality breaks through the scope of its classical immune regulation, expanding its application in the reproductive field by regulating metabolic coupling between granulosa cells and oocytes.

In male reproduction, ICA protects the spermatogenic microenvironment and stabilizes the blood–testis barrier by regulating the balance between oxidative stress through Nrf2 pathway activation and inflammation via inhibition of the NLRP3 inflammasome and pro-inflammatory cytokine release, thereby providing robust support for normal spermatogenesis. Meanwhile, it can repair the homeostasis of the “reproductive–immune–vascular” axis by alleviating endothelial injury, inhibiting platelet activation, and reducing vascular inflammation, offering an idea for the treatment of vascular ED and fully reflecting its regulatory potential in the male reproductive system.

Notably, the multi-target and multi-pathway regulatory characteristics of ICA have duality. On one hand, this characteristic enables it to address the complex pathological microenvironment of reproductive system diseases, which typically involve interactions among inflammation, immunity, and metabolism, thereby conferring unique advantages compared to single-target drugs. On the other hand, this complexity increases the difficulty of elucidating its precise mechanisms of action. Current studies mostly focus on a single target or pathway, and the synergistic network between different regulatory mechanisms remains unclear, which needs further exploration with the help of systems biology and other methods.

In conclusion, ICA demonstrates broad application prospects in the treatment of reproductive system diseases by regulating the reproductive–immune axis and related metabolic pathways. However, further in-depth studies are still needed to clarify its detailed regulatory network, optimize its pharmacokinetic characteristics, and verify its clinical efficacy and safety. Only by clarifying the above research priorities can we gradually promote the clinical transformation of ICA and provide new therapeutic options for clinical reproductive medicine.

## Figures and Tables

**Table 1:** Mechanisms of ICA in modulating the reproductive–immune axis.

Axis Level and Core Pathway	Molecular Actions of ICA	Primary Cellular and Molecular Targets	Integrated Immunological and Reproductive Outcomes	Related Experimental Models	Refs
Immune Modulation and Inflammatory Control					
Suppression of Innate Immune and Pro-inflammatory Signaling	Inhibits NF-κB activation (IκBα degradation, p65 nuclear translocation); suppresses MAPK pathway (p38, JNK, ERK phosphorylation); scavenges ROS.	Macrophages, tissue-resident immune cells, endothelial cells, Leydig cells, granulosa cells.	Immune Outcome: ↓ TNF-α, IL-1β, IL-6, COX-2. Reproductive Outcome: ↓ Testicular/ovarian inflammation; ↑ survival of Leydig/granulosa cells; ↑ testosterone synthesis (via upregulation of CYP11A1, 3β-HSD).	DEHP-induced testicular injury/dysfunction; hypertensive ED (SHR model); autoimmune orchitis; endometriosis (predicted).	[49,51,105,115,116]
Inhibition of Inflammasome Activation	Suppresses NLRP3 inflammasome assembly and caspase-1 cleavage.	Macrophages, ovarian granulosa cells, renal cells.	Immune Outcome: ↓ Maturation and secretion of IL-1β; ↓ pyroptosis. Reproductive Outcome: ↓ Apoptosis of ovarian granulosa cells; preservation of follicular reserve.	CTX-induced POI; pZP3-induced autoimmune POI; SLE-associated inflammation (MRL/lpr lupus nephritis model).	[60,71,120]
Regulation of Adaptive Immune Balance	Inhibits IL-17A production and IL-6 expression; suppresses MEK-ERK signaling downstream of IL-17; modulates Th1/Th17/Treg cell differentiation.	Th17 cells, Treg cells, splenic T cell subsets, ovarian tissue immune microenvironment.	Immune Outcome: ↓ Serum IL-17A; ↑ proportion of CD4^+,^ CD25^+,^ Foxp3^+,^ Treg cells. Reproductive Outcome: ↓ Ovarian lymphocyte infiltration; ↑ serum AMH and E2; ↓ FSH and LH; protects primordial follicles; improves folliculogenesis.	CTX-induced POI; autoimmune POI (pZP3 mouse model); potential application in PCOS-related inflammation.	[66,71,120]
Direct Protection and Functional Enhancement of Reproductive Tissues					
Activation of Cytoprotective and Antioxidant Pathways	Activates Nrf2 signaling (promotes nuclear translocation) and upregulates HO-1, NQO-1; upregulates Sirt1 expression; increases SOD activity; decreases MDA content.	Sertoli cells (TM4 line), granulosa cells, testicular/ovarian somatic cells.	Reproductive Outcome: ↑ Cellular antioxidant defense; ↓ oxidative stress and senescence (↓ SA-β-gal); ↑ function of supportive cells (↑ GDNF, PLZF in Sertoli cells). Immune Interface: Creates an anti-inflammatory tissue microenvironment.	D-galactose-induced senescence in Sertoli cells; naturally aging rat testis mode; autoimmune ovarian injury model.	[71,93]
Regulation of Hormonogenesis and Germ Cell Support	Upregulates steroidogenic enzymes (CYP11A1, 3β-HSD, 17β-HSD) and SF-1; activates ERα/Nrf2 crosstalk; modulates serum FSH, LH, E2, AMH levels.	Leydig cells, Sertoli cells, ovarian follicles, granulosa cells.	Reproductive Outcome: ↑ Testosterone and estradiol synthesis; ↑ sperm count and viability; ↑ primordial and Graafian follicle counts; restores estrous cyclicity.	Age-related testicular dysfunction; CTX-induced ovarian damage/POI; DEHP-induced testicular injury.	[49,51,66,93,105,116]
Improvement in Vascular and Erectile Function	Selectively inhibits PDE5 enzyme (IC_50_ = 0.432 µM), elevating cGMP levels; improves endothelial function via anti-inflammatory/antioxidant actions.	Corpus cavernosum smooth muscle and endothelial cells, platelets, EPCs.	Reproductive Outcome: Promotes smooth muscle relaxation; improves penile hemodynamics and erectile function. Immune/Vascular Interface: ↓ Endothelial activation and platelet aggregation.	Spontaneously Hypertensive Rat (SHR) model of ED; in vitro human platelet PDE5 activity study.	[121]
Systemic and Interface-Level Modulation					
Tissue-Selective Estrogenic Activity	Activates non-genomic ERα signaling (phosphorylation of Ser118/167, MAPK/ERK, PI3K/Akt) without genomic ERE-driven transcription.	Bone (osteoblasts/osteoclasts), vascular endothelium, brain.	Systemic/Interface Outcome: ↑ BMD in OVX rats; vascular and neuroprotection. Reproductive Health Relevance: Indirect support via maintaining systemic and vascular homeostasis, without uterine proliferation.	OVX rat model of postmenopausal osteoporosis; immature mouse uterine hyperplasia model (showing lack of proliferation).	[55,122]
Synergistic and Potential Regulatory Mechanisms	May enhance GR-mediated repression of NF-κB target genes; modulation of pathways affecting oocyte quality (e.g., mevalonate pathway).	GR DNA-binding domain, NF-κB response elements; oocyte cytoskeleton (based on related pathways).	Theoretical Interface: Potentiation of anti-inflammatory gene repression; potential improvement in oocyte cytoskeletal dynamics and quality in aging.	General inflammation-driven reproductive disorders; ovarian aging (indirect mechanistic link).	[104,123]

↑ means increase, ↓ meanst decrease. Reactive Oxygen Species: ROS, Nuclear Factor kappa-B: NF-κB, Inhibitor of NF-κB: IκBα, Mitogen-Activated Protein Kinase: MAPK, c-Jun N-Terminal Kinase: JNK, Extracellular Signal-Regulated Kinase: ERK, Cyclooxygenase-2: COX-2, Phthalate: DEHP, Spontaneously Hypertensive Rat: SHR, NOD-Like Receptor Family Pyrin Domain Containing 3: NLRP3, Interleukin: IL, Premature Ovarian Insufficiency: POI, zona pellucida 3: pZP3, Systemic Lupus Erythematosus: SLE, Murine Lymphoma cell line: MRL/lpr, T helper: Th, regulatory T: Treg, anti-Müllerian hormone: AMH, estradiol: E2, polycystic ovary syndrome: PCOS, Nuclear factor erythroid 2-related factor 2: Nrf2, Heme Oxygenase-1: HO-1, NAD(P)H: Quinone Oxidoreductase 1: NQO-1, Sirtuin 1: Sirt1, Superoxide Dismutase: SOD, Malondialdehyde: MDA, Senescence-Associated Beta-galactosidase: SA-β-gal, Glial cell line-Derived Neurotrophic Factor: GDNF, Promyelocytic Leukemia Zinc Finger: PLZF, Cytochrome P450 Family 11 Subfamily A Member 1: CYP11A1, 3β-Hydroxysteroid Dehydrogenase: 3β-HSD, 17β-Hydroxysteroid Dehydrogenase: 17β-HSD, Steroidogenic Factor 1: SF-1, Estrogen Receptor alpha: ERα, Phosphodiesterase 5: PDE5, cyclic Guanosine Monophosphate: cGMP, endothelial progenitor cells: EPCs, erectile dysfunction: ED, Estrogen Response Element: ERE, Bone Mineral Density: BMD, Ovariectomized: OVX, Glucocorticoid Receptor: GR, Deoxyribonucleic Acid: DNA.

## Data Availability

No new data were created or analyzed in this study.

## Abbreviations

3β-HSD	3β-Hydroxysteroid Dehydrogenase
8-IPF	8-Isopentenylflavone
17β-HSD	17β-Hydroxysteroid Dehydrogenase
AMH	Anti-Müllerian Hormone
BMD	Bone Mineral Density
cGMP	Cyclic Guanosine Monophosphate
CMS	Chronic Mild Stress
COX-2	Cyclooxygenase-2
CYP11A1	Cytochrome P450 Family 11 Subfamily A Member 1
DCs	Dendritic Cells
DEHP	Phthalate
DNA	Deoxyribonucleic Acid
dNK	Decidual Natural Killer
E2	Estradiol
EAE	Experimental Autoimmune Encephalomyelitis
ED	Erectile Dysfunction
EMPs	Endothelial Microparticles
EPCs	Endothelial Progenitor Cells
ERα	Estrogen Receptor Alpha
ERE	Estrogen Response Element
ERK	Extracellular Signal-Regulated Kinase
GC	Glucocorticoid
GDNF	Glial Cell Line-Derived Neurotrophic Factor
GR	Glucocorticoid Receptor
HO-1	Heme Oxygenase-1
HPLC	High-Performance Liquid Chromatography
ICA	Icariin
IL	Interleukin
IL-4	Interleukin-4
IκBα	Inhibitor of NF-κB
JNK	c-Jun N-Terminal Kinase
MAPK	Mitogen-Activated Protein Kinase
MDA	Malondialdehyde
MRL/lpr	Murine Lymphoma Cell Line
NF-κB	Nuclear Factor Kappa-B
NLRP3	NOD-Like Receptor Family Pyrin Domain Containing 3
NQO-1	NAD(P)H: Quinone Oxidoreductase 1
Nrf2	Nuclear Factor Erythroid 2-Related Factor 2
OVX	Ovariectomized
PBMCs	Peripheral Blood Mononuclear Cells
PCOS	Polycystic Ovary Syndrome
PDE5	Phosphodiesterase 5
PGE2	Prostaglandin E2
PLZF	Promyelocytic Leukemia Zinc Finger
POI	Premature Ovarian Insufficiency
PPARs	Peroxisome Proliferator-Activated Receptors
pZP3	Zona Pellucida 3
RIF	Recurrent Implantation Failure
ROS	Reactive Oxygen Species
RSA	Recurrent Spontaneous Abortion
SA-β-gal	Senescence-Associated Beta-Galactosidase
SF-1	Steroidogenic Factor 1
SHR	Spontaneously Hypertensive Rat
Sirt1	Sirtuin 1
SLE	Systemic Lupus Erythematosus
SOD	Superoxide Dismutase
Th	T Helper
Treg	Regulatory T
